# ACTL6A regulates the Warburg effect through coordinated activation of AP-1 signaling in head and neck squamous cell carcinoma

**DOI:** 10.1101/2025.09.02.671886

**Published:** 2025-09-04

**Authors:** Mehri Monavarian, Alistaire R. Sherman, Imran A. Mohammad, Sainiteesh Maddineni, Mao Zhang, Joseph C. Wu, Katrin F. Chua, John Sunwoo, Andrey Finegersh

**Affiliations:** 1 Department of Otolaryngology, Stanford University, Palo Alto, CA; 2 Department of Medicine, Stanford University, Palo Alto, CA; 3 Veterans Affairs Palo Alto Health Care System, Palo Alto, CA

## Abstract

ACTL6a is an essential component of SWI/SNF and expressed on the chromosome 3q26 cytoband, which is amplified in head and neck squamous cell carcinomas (HNSCC). While ACTL6A is emerging as an oncogene, its role as a treatment target and mechanisms of transcription factor induction remain unknown. Here, we show that ACTL6A expression is a mediator of the Warburg effect, with ACTL6A knockdown inducing mitochondrial dependency and significantly decreasing levels of aerobic glycolysis. These effects lead to near complete attenuation of hypoxic cell growth by blunting induction of HIF1α and HIF2α protein expression. They also sensitize treatment resistant HNSCC cells to the tumor killing effects of the complex I inhibitor IACS-010759 *in vivo*. Using ATAC-seq, we identify ACTL6A as a mediator of chromatin accessibility of AP-1 transcription factor sites and find that it regulates upstream MAPK signaling through induction of Ras and Galectin-1. These effects sensitize ACTL6A over-expressing cells to inhibition of glycolysis by MEK inhibitors. Our results link SWI/SNF subunit amplification with potentiation of MAPK signaling in HNSCC and provide a novel mechanism by which cancer cells drive aerobic glycolysis and reduce mitochondrial dependency. We leverage these findings to propose treatment strategies for hypoxic tumors with SWI/SNF subunit amplifications.

## Introduction

Head and neck squamous cell carcinoma (HNSCC) is the sixth most common malignancy^1^ and treatment confers significant morbidity and functional deficits for survivors^2^. Treatment paradigms have remained unchanged for several decades and involve platinum-based systemic therapies, radiation, surgery, with perioperative immunotherapy recently gaining approval despite no benefit to overall survival^3^. HNSCC survival has only minimally improved over several decades^4^ and HPV-negative HNSCC has a 5-year overall survival around 50%^5,6^. Moreover, oral and pharyngeal carcinomas were the only cancers to have declining overall survival and increasing incidence on the 2025 North American Association of Central Cancer Registries report^7^, suggesting an urgent need to develop treatments for this malignancy.

Like other solid tumors, HNSCC shows increased dependence on glycolysis^8^, with increasing rates of glycolysis in tumors leading to worse survival and decreased immune cell infiltration^9^. Reliance on glycolysis also contributes to hypoxic tumor growth, which allows tumors to evade immune responses and contributes to chemotherapy and radiation resistance^10,11^. Tumor hypoxia was recently used to stratify patients for de-escalation of HPV-associated HNSCC, with hypoxic tumors needing more intensive treatment to achieve similar overall survival^12^. Additionally, inhibition of oxidative phosphorylation contributes to cell survival and growth by decreasing production of reactive oxygen species (ROS) and shunting glycolytic metabolites into anabolic pathways^13^. The metabolic shift toward aerobic glycolysis in cancer cells is a phenomenon described by Otto Warburg in the 1920s^14^; however, mechanisms of metabolic reprogramming in tumors remain poorly understood and have yet to be clinically exploited in HNSCC. Regulators of glycolysis in HNSCC are being studied and have been proposed as therapeutic targets, including HSP90^15^ and HGF^15^, indicating a potential role in patient care.

Copy number amplification of chromosome 3q26 occurs in up to 75% of HNSCCs^17^ and is associated with over-expression of several oncogenic drivers^18^. One of these oncogenes, ACTL6A, is a component of the SWI/SNF (BAF) complex, which is an ATP-dependent chromatin remodeling complex that is critical in the development and maintenance of cell fate^19^, RNA polymerase II mediated gene transcription^20^, and DNA repair^21^. ACTL6A has been implicated in driving TEAD/YAP activation in HNSCC to maintain a poorly differentiated phenotype^22–24^. These effects are mediated by increased SWI/SNF chromatin occupancy with ACTL6A expression, as evidenced by Chang et al. (2021), who titrated levels of ACTL6A within the HNSCC cell line FaDu to show that ACTL6A levels are directly tied to SWI/SNF binding at genes necessary for SCC tumor initiation and maintenace^23^. Recent studies in other cancers have linked ACTL6A expression with maintenance of metabolic phenotypes, including activation of glutathione synthesis and inhibition of ferroptosis in gastric carcinoma^25^ and stimulation of glycolysis through FSH and PGK1 in ovarian cancer^21^.

While ACTL6A amplification has been linked to increased SWI/SNF chromatin occupancy and YAP activation in HNSCC, the functional consequences of its expression and role as a therapeutic target are not defined. This study identifies ACTL6A as a key regulator of aerobic glycolysis and hypoxic cell growth in HNSCC, defines a novel association between ACTL6A expression and AP-1 signaling, and establishes a synthetic lethality strategy to drive tumor killing in a treatment resistant HNSCC cell line.

## Results

### ACTL6A levels regulate aerobic glycolysis in HPV (−) HNSCC

**Figure 1.**
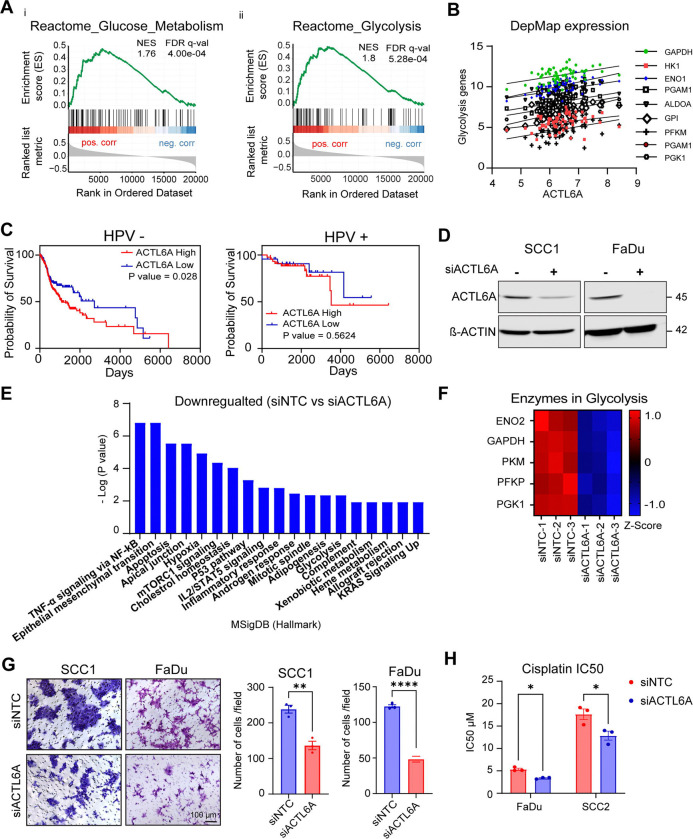
ACTL6A is associated with increased aerobic glycolysis in HNSCC A) Enrichment plots for glucose metabolism (i) and glycolysis (ii) Reactome gene sets showing correlation with ACTL6A expression in TCGA HNSCC patients. NES: Normalized enrichment score, FDR: False Discovery Rate B) Correlation analysis between ACTL6A and glycolysis genes in 62 HNSCC cell lines from DepMap. C) Survival analysis using log-rank test in HPV (−) (right) and HPV (+) (left) HNSCCs from TCGA D) Western blot of ACTL6A and β-actin levels in SCC1 and FaDu cells following transient ACTL6A knockdown. E) Most significantly down-regulated Hallmark pathways in SCC1 cells after siACTL6A. F) Heatmap of glycolysis gene expression in siACTL6A and control SCC1 cells using Z-scores calculated from RNA-seq data. G) Representative images (left) and quantitation (right) of SCC1 and FaDu cells following transient ACTL6A knockdown after 24 hour incubation in fibronectin coated transwell membrane. (n=3). All data are mean ± SEM, ** p < 0.01, **** p <0.0001, unpaired t test. Scale bar: 100 μm H) IC50 of cisplatin treatment following transient ACTL6A knockdown in indicated cells after 72 hour drug incubation. (n=3). The data are mean ± SEM, * p < 0.05, multiple unpaired t tests.

We used The Cancer Genome Atlas (TCGA) to study correlations between ACTL6A expression within the HNSCC RNA-seq dataset and metabolic pathways using the Reactome gene set enrichment analysis (GSEA)^26–28^. GSEA showed significant correlations between glucose metabolism and glycolysis with ACTL6A expression in HNSCC tumors (p<0.001) (Fig. 1A). To further assess if glycolysis gene expression is correlated with ACTL6A levels across different mutational backgrounds, we performed a correlational analysis on 62 cell lines derived from oral cavity and larynx SCC from DepMap^29^. We found significant positive correlations between ACTL6A and expression of all glycolysis genes (Fig. 1B and table S1). We then stratified tumors by HPV status in TCGA and found that high expression of ACTL6A correlated with lower survival in HPV (−) but not HPV (+) HNSCC (Fig. 1C). Based on this, we performed RNA-seq on the HPV (−) HNSCC cell line SCC1 following transient ACTL6A knockdown to test the hypothesis that ACTL6A levels are mechanistically linked to expression of glycolysis genes (Fig. 1D). We used Molecular Signatures Database (MSigDB) hallmark gene set and found down-regulation of hypoxia, glycolysis, and KRAS pathways in the ACTL6A depleted samples (Fig. 1E). Additionally, there was significant down-regulation of the glycolysis genes ENO2, GAPDH, PKM, PFKP, and PGK1 (Fig. 1F).

ACTL6A knockdown was previously shown to down-regulate cell proliferation and spheroid formation in HNSCC^22^. We examined effects of ACTL6A knockdown on additional oncogenic cell phenotypes associated with glycolysis, including cell motility^30^ and cisplatin resistance^31^. Using a transwell migration assay, we found that siRNA knockdown of ACTL6A led to a significant reduction in cell migration in two independent HPV-negative cell lines, SCC1 and FaDu (>60% and >40% reduction, respectively) (Fig. 1G). We then measured the IC50 of cisplatin in FaDu and UD-SCC2 cells, an additional HPV-negative cell line, and found significantly increased sensitivity to cisplatin in ACTL6A knockdown compared to control cells (Fig. 1H). These findings are consistent with down-regulation of the epithelial mesenchymal transition (p < 0.001) and xenobiotic metabolism (p < 0.05) pathways in ACTL6A knockdown cells observed in our GSEA (Fig. 1E).

### ACTL6A regulates mitochondrial dependency

**Figure 2.**
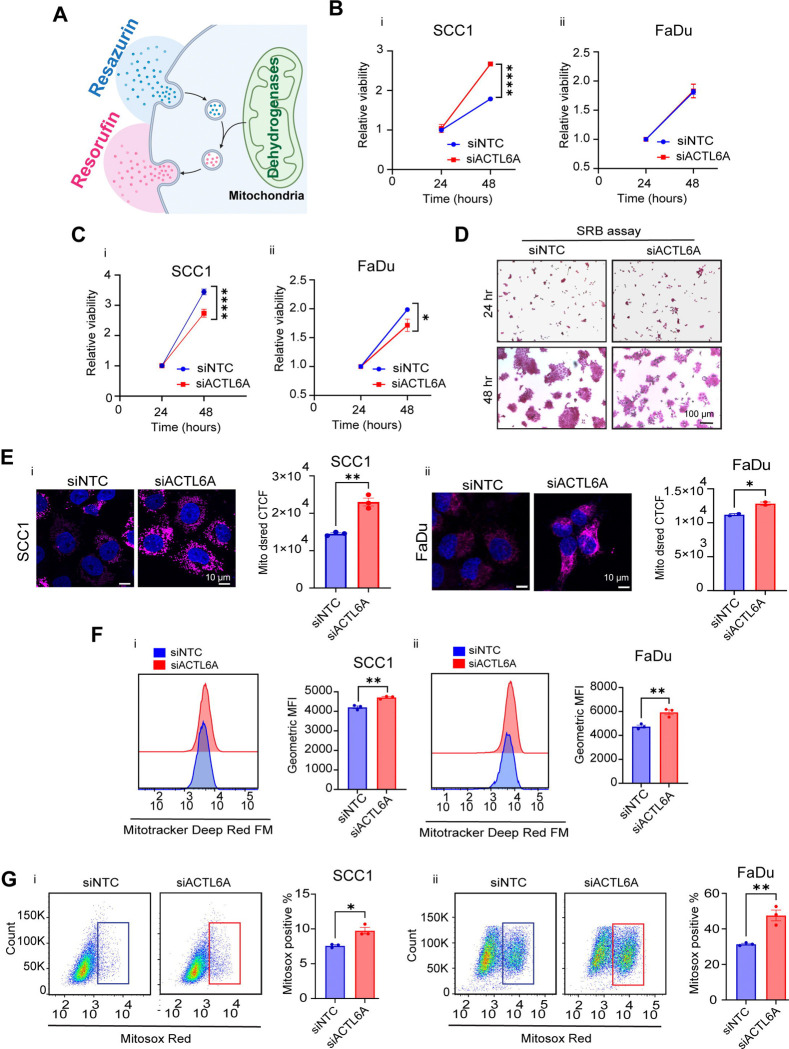
Mitochondrial function is enhanced by ACTL6A knockdown. A) Schematic of sesazurin assay showing the reduction of resazurin to resorufin by mitochondria in live cells. B) Relative viability of SCC1 (i) and FaDu (ii) cells upon ACTL6A knockdown measured by resazurin assay after 24- and 48-hours incubation. (n=3). All data are mean ± SEM, ns p > 0.05, **** p <0.0001, two-way ANOVA followed by Sidak’s multiple comparisons test. C) Relative viability of SCC1 (i) and FaDu (ii) cells upon ACTL6A knockdown measured by SRB assay after 24- and 48-hours incubation. (n=3). All data are mean ± SEM, * p < 0.05, **** p <0.0001, two-way ANOVA followed by Sidak’s multiple comparisons test. D) Representative images of SCC1 cells stained with SRB after transient ACTL6A knockdown at indicated time points. Scale bar: 100 μm E) Representative confocal images and quantitation of MitoDsRed (magenta) normalized to the number of nuclei stained with DAPI (blue) in SCC1 (i) and FaDu (ii) cells following transient ACTL6A knockdown. (n=3 in SCC1 and n=2 in FaDu). Data are mean ± SEM, * p < 0.05, ** p < 0.01, unpaired t tests. Scale bar: 10μm. F) Representative histogram and quantitation of Geometric Mean Fluorescence (GMF) of Mitotracker Deep Red FM in SCC1 (i) and FaDu (ii) analyzed by flowcytometry after 72 hours transient ACTL6A knockdown. (n=3), Data are mean ± SEM, ** p < 0.01, unpaired t test. G) Representative pseudo color graphs from flowcytometry analysis and quantitation of percentage of Mitosox positive in SCC1 (i) and FaDu (ii) cells following transient ACTL6A knockdown. N=3. Data are mean ± SEM, * p < 0.05, ** p < 0.01, unpaired t tests.

There are no prior studies on how ACTL6A levels influence mitochondrial properties. In our initial experiments, we performed a resazurin based cell viability assay, which uses mitochondrial reduction of resazurin to resofurin as a correlate to cell number and viability^32^ (Fig. 2A). Although, we observed decreased cell proliferation following ACTL6A knockdown compared to control cells on microscopy (Fig. 2D), the resazurin assay either showed increased or no change in cell proliferation after the knockdown in SCC1 and FaDu respectively (Fig. 2B). Thus, we decided to assess cell proliferation by Sulforhodamine B (SRB), which measures the total protein mass in cells^33^. The SRB assay showed modest but significantly decreased proliferation at 48 hours in both SCC1 and FaDu cells after ACTL6A knockdown (mean relative viability of 2.7 in siACTL6A versus 3.4 in siNTC p value < 0.0001 in SCC1 and 1.71 versus 1.98 with p value = 0.016 in FaDu) (Fig. 2C,D). Based on this discrepancy between resazurin and SRB assays, we hypothesized that ACTL6A affects mitochondrial dependency. We initially assessed mitochondrial mass by transducing SCC1 and FaDu cells with the MitoDsRed plasmid then transiently knocked down ACTL6A. We observed significantly increased intensity of MitoDsred after ACTL6A knockdown in both cell lines, indicative of enhanced mitochondrial density and mass (Fig. 2E). We then used Mitotracker deep red (MTDR) staining to study alterations in mitochondrial potential and activity in SCC1 and FaDu cells and found significantly increased MTDR staining after ACTL6A knockdown (Fig. 2F). These findings suggest down-regulation of ACTL6A enhances mitochondrial density and potential in HNSCC cells.

Mitochondria are the main source of reactive oxygen species (ROS), which are generally cytotoxic to cancer cells and regulated through oncogenic redox homeostasis pathways^34^. We tested whether increased mitochondrial activity in cells with ACTL6A knockdown could lead to increase superoxide generation using MitoSOX red staining followed by flow cytometry. We observed a significant increase in MitoSOX intensity after ACTL6A knockdown in both SCC1 and FaDu cells (Fig. 2G).

### ACTL6A expression alters HNSCC cell metabolism

**Figure 3.**
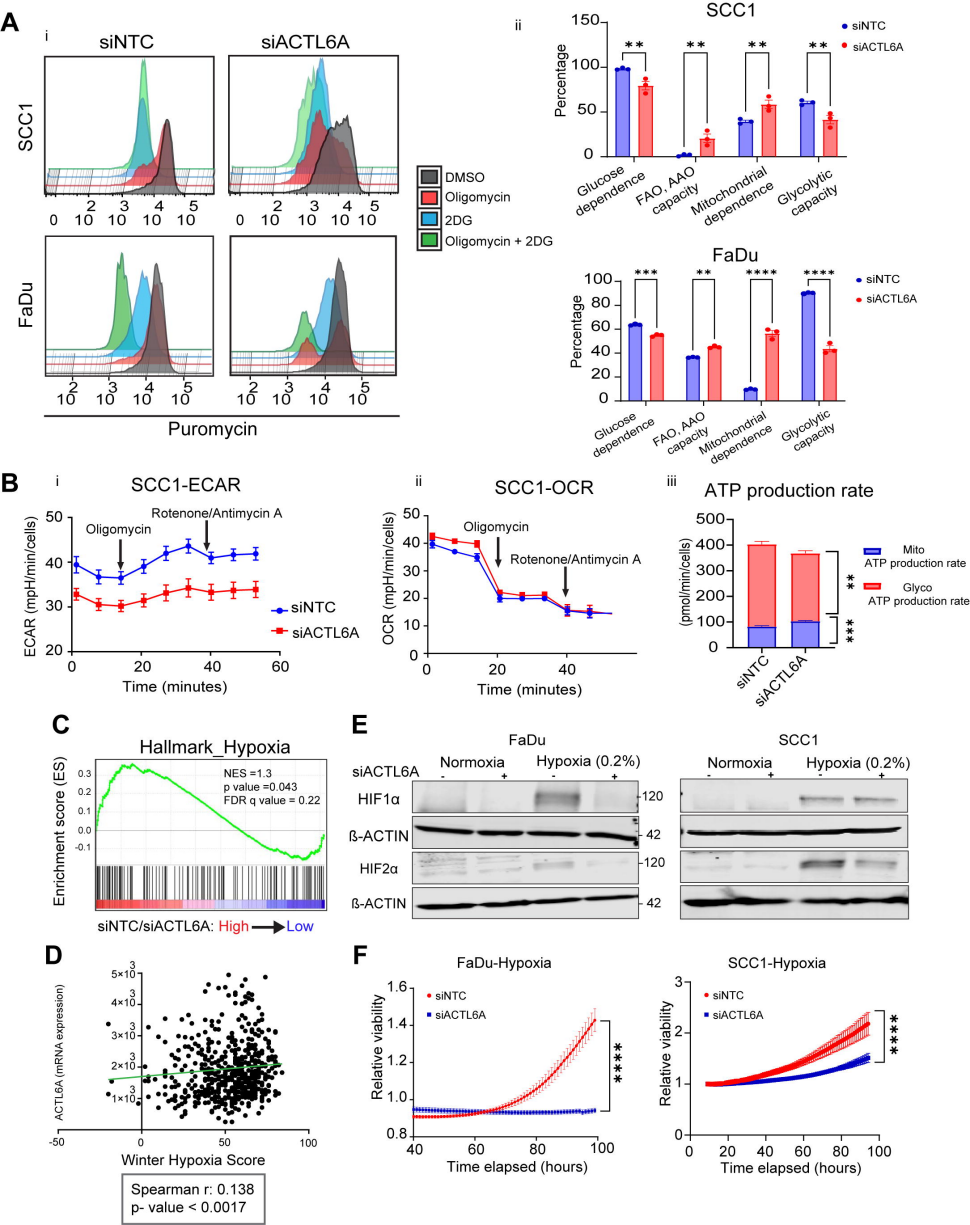
ACTL6A knockdown increases mitochondrial dependency and reduces aerobic glycolysis in HNSCC cells. A) Histogram plots of puromycin fluorescence intensity (i) and quantitation (ii) of percentage of mitochondrial dependence, glycolytic capacity, glucose dependence, and fatty acid/amino acid oxidation (FAO, AAO) capacity from SCENITH assay (flowcytometry based) in SCC1 (upper panel) and FaDu cells (lower panel) following ACTL6A knockdown. N=3. Data are mean ± SEM, B) Representative extracellular acidification rate (ECAR) (i), oxygen consumption rate (ii), ATP production rate (iii) from SCC1 after transient ACTL6A knockdown using Seahorse XF Real-Time ATP Rate assay. N=2. ** p < 0.01, *** p <0.0001, two-way ANOVA followed by Sidak’s multiple comparisons test. C) Enrichment plot for Hallmark hypoxia gene set using RNA-seq from SCC1 cells following transient ACTL6A knockdown analyzed with GSEA. N=3. Cut off for p value < 0.05, FDR q value < 0.25. E) Representative Western blots of HIF1α, HIF2α, and β-actin in FaDu (left) and SCC1 cells (right). Following transient ACTL6A knockdown, the cells were incubated either in normoxia or 0.2% hypoxia for 24 hours and protein expression was measured using western blot. F) Correlation analysis between ACTL6A mRNA expression and Winter hypoxia score in HNSCC tumors from TCGA. G) Relative viability of FaDu (left) and SCC1 (right) cells following ACTL6A transient knockdown was measured at indicated time points in 0.2% hypoxia using xCELLignece system. N=6. **** p <0.0001, two-way ANOVA followed by Sidak’s multiple comparisons test.

To characterize how ACTL6A influences HNSCC cell metabolism, we performed a flow cytometry-based assay called SCENITH (single-cell energetic metabolism by profiling translation inhibition) that uses protein translation as a correlate of energy consumption within cells^35^. We treated the cells with glycolysis or oxidative phosphorylation inhibitors including 2-deoxy-d-glucose (2DG) and oligomycin, respectively, or both to block total ATP production. Next, we incubated the cells with puromycin and stained with anti-puromycin antibody to assess the changes in protein synthesis in SCC1 and FaDU cells following ACTL6A knockdown (Fig. 3A). These data showed that upon addition of oligomycin (Fig 3Ai, red), MFI (mean Fluorescence intensity) of puromycin decreased more significantly in siACTL6A compared with siNTC, which is indicative of higher mitochondrial dependency and lower glycolytic capacity in siACTL6A cells (Fig. 3Aii). However, 2DG treatment (Blue color) led to a more significant reduction in MFI of puromycin in siNTC compared with siACTL6A in both cell lines, suggestive of higher glucose dependency and lower fatty acid and amino acid oxidation capacity (FAO and AAO) in siNTC cells (Fig. 3Aii).

Additionally, we performed a Seahorse XF Real-Time ATP Rate assay in SCC1 cells following transient ACTL6A knockdown to estimate ATP production from mitochondria and glycolysis by measuring extracellular acidification (ECAR) and oxygen consumption rate (OCR) in media upon sequential treatment with oligomycin (Complex V inhibitor) and rotenone/antimycin A (complex I and III inhibitors). The ATP rate assay revealed diversion of glycolysis to oxidative phosphorylation while total ATP production did not change with ACTL6A knockdown (Fig. 3B), which was consistent with SCENITH assay. These findings indicate that high ACTL6A levels up-regulate glycolysis and down-regulate oxidative phosphorylation and fatty acid oxidation, consistent with decreasing mitochondrial dependency.

### ACTL6A-deficient cells have reduced ability to respond to hypoxia

Increased levels of aerobic glycolysis and decreased reliance on oxidative metabolism are adaptations that promote hypoxic tumor growth^36^. Hypoxia is also a critical survival mechanism of HNSCC tumors and drives treatment resistance^11,12^. Based on our data that ACTL6A regulates cell metabolism and mitochondrial dependency, we hypothesized that ACTL6A expression is required for hypoxic cell growth. We first explored differential expression of the genes that make up the Buff hypoxia gene set^37^ using our SCC1 RNA-seq data, which showed downregulation of a majority of hypoxia genes in ACTL6A knockdown compared to control cells (Extended Data Fig. 1). Furthermore, Hallmark GSEA for hypoxia was up-regulated in siNTC relative to siACTL6A cells (Fig. 3C). We then used TCGA to identify whether these changes occurred within HNSCC tumors and found that ACTL6A expression was positively correlated with Winter hypoxia score in patients with HNSCC^38^ (Fig. 3D).

The hypoxia inducible factors HIF1α and HIF2α are genomic regulators of hypoxic response whose expression is regulated through post-translational modifications and degradation under normoxic conditions. To study mechanisms by which ACTL6A expression may influence hypoxic responses, we measured HIF protein expression in FaDu and SCC1 following siACTL6A in normoxia or hypoxia (0.2% O2) for 24 hours. We found that siACTL6A significantly abrogated induction of HIF2α protein expression under hypoxia in both cell lines (Fig. 3E). We also observed similar response for HIF1α in FaDu cells (Fig. 3E). Next, we cultured FaDu and SCC1 in hypoxic conditions using xCELLignece to assess the effect of ACTL6A levels on hypoxic cell growth. We found that siACTL6A significantly attenuated growth in both cell lines, essentially preventing the ability of either cell line to proliferate at low oxygen levels (Fig. 3F). These results suggest that ACTL6A regulates hypoxia in HNSCC by stabilizing HIF proteins under hypoxic conditions and inducing expression of hypoxia responsive genes.

### Targeting ACTL6A with mitochondrial inhibitors as a synthetic lethal strategy for treatment resistant HNSCC tumors

**Figure 4.**
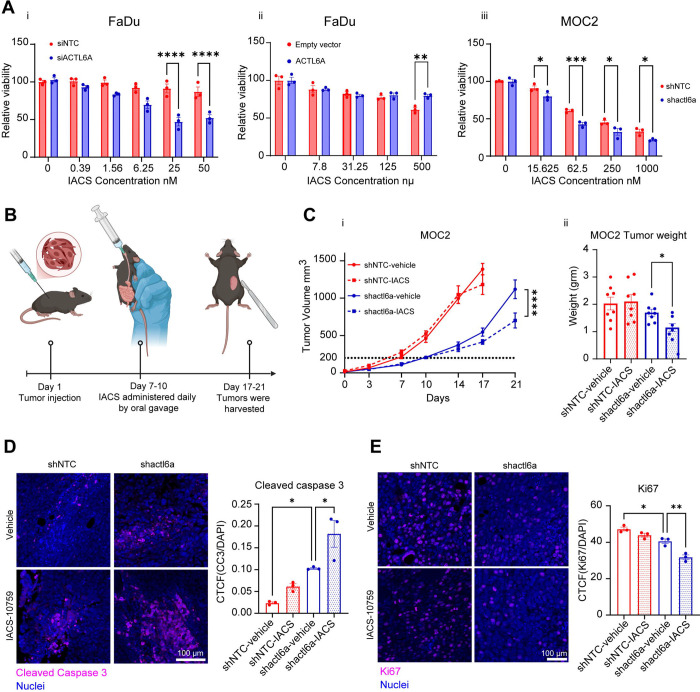
ACTL6A knockdown sensitizes HNSCC cells to treatment with the complex I inhibitor IACS-010759. A) Relative viability of FaDu cells following ACTLA6 transient knockdown (i), or ACTL6A overexpression (ii), and (iii) MOC2 cells following ACTL6A stable knockdown was measured using SRB assay after incubation with either DMSO or indicated concentrations of IACS-010759 for 5 days. N=3. * p < 0.05, ** p < 0.01, *** p <0.0001, **** p <0.0001, two-way ANOVA followed by Sidak’s multiple comparisons test. B) Schematic of animal study showing the timeline for injection of tumor cells and administration of IACS-010759. C) (i) Tumor volume was measured using calipers at indicated time points after injection with MOC2 cells expressing shACTL6A or control vector. When tumor size reached 200 mm^3^ (dashed line), mice were treated with either control vehicle or IACS-010759 by oral gavage. N=9–10/group, ns p > 0.05, ****p < 0.0001, two-way ANOVA followed by Sidak’s multiple comparisons test. (ii) Tumor weight was measured at study endpoint. ns p > 0.05, * p < 0.05, unpaired t tests. D) Representative confocal images from tumor tissue stained for cleaved caspase 3 (cc3) (magenta) and DAPI (blue) (left) and quantification (right) of corrected total cell fluorescence of cc3 normalized to DAPI in indicated samples. N=3. * p < 0.05, one-way ANOVA followed by Tukey’s multiple comparisons test. Scale bar: 100μm E) Representative confocal images from mice tissue stained for Ki67 (magenta) and DAPI (blue) (left) and quantification (right) of corrected total cell fluorescence of Ki67 normalized to DAPI in indicated samples. N=3. * p < 0.05, ** p < 0.01, one-way ANOVA followed by Tukey’s multiple comparisons test. Scale bar: 100μm

Our data suggest that ACTL6A depletion in HNSCC cells triggers a switch from aerobic glycolysis to mitochondrial dependence. We therefore hypothesized that decreasing ACTL6A levels could sensitize cells to mitochondrial inhibitors. We used IACS-010759, a potent inhibitor of oxidative phosphorylation that targets mitochondrial complex I and has been in clinical trials for solid tumors^39^ to assess chemosensitivity of HNSCC cell lines to mitochondrial inhibitors. We assessed IC50 to IACS-010759 after ACTL6A knockdown in FaDu cells, which showed significantly enhanced sensitivity to low doses of drug at 25 and 50 nM in siACTL6A cells (Fig. 4Ai). Conversely, FaDu cells over-expressing ACTL6A were significantly more treatment resistant to IACS-010759 relative to control cells, even at high drug concentrations of 500 nM (Fig. 4Aii). We then assessed sensitivity of MOC2, a mouse oral cavity carcinoma cell line induced by carcinogen exposure, which are highly proliferative and resistant to cisplatin, radiation, immunotherapy, and combination therapies^40,41^. We generated a stable ACTL6A knockdown using shACTL6A with a ~50% reduction in ACTL6A levels (Extended Fig. 2). We found shACTL6A MOC2 cells had significantly increased sensitivity to IACS-010759 relative to control cells across a range of drug concentrations (Fig. 4Aiii).

Based on the synergistic effects of ACTL6A knockdown and IACS-010759 treatment *in vitro*, we investigated the effect of this combined treatment on tumor growth *in vivo.* We performed a heterotopic tumor model using control (shNTC) or ACTL6A-depleted (shACTL6A) MOC2 cells subcutaneously injected into B6J.Rag2 Il2rg double KO mice, which allowed us to study drug effects in isolation without tumor-immune responses. Mice were treated with either vehicle or 10 mg/kg IACS-010759 by oral gavage starting when average tumor size reached 200 mm^3^ (Fig. 4B). After treatment onset, there was a significant reduction in tumor size with IACS-010759 treatment compared to vehicle in ACTL6A-depleted tumors while no change in tumor volume was observed in control tumors (Fig. 4Ci). Tumors were harvested upon reaching 1000 mm^3^ in the control group and endpoint measurement of tumor weight revealed similar results, with IACS-010759 treatment leading to significantly lower tumor weight only in ACTL6A-depleted tumors (fig. 4Cii). To identify whether this was primarily an effect of reduced proliferation or tumor cell death, we measured cleaved caspase 3 (CC3) and Ki67 expression as a marker of apoptosis and proliferation, respectively on explanted tumors. Immunofluorescence staining showed both a significant increase in apoptosis and a reduction proliferation with IACS-010759 treatment only in the ACTL6A-depleted tumors (Fig. 4D,E). These data indicate targeting ACTL6A in combination with inhibitors of oxidative phosphorylation or mitochondrial function may be a treatment strategy for treatment resistant HNSCC.

### ACTL6A modulates chromatin accessibility at AP-1 transcription factor sites

**Figure 5.**
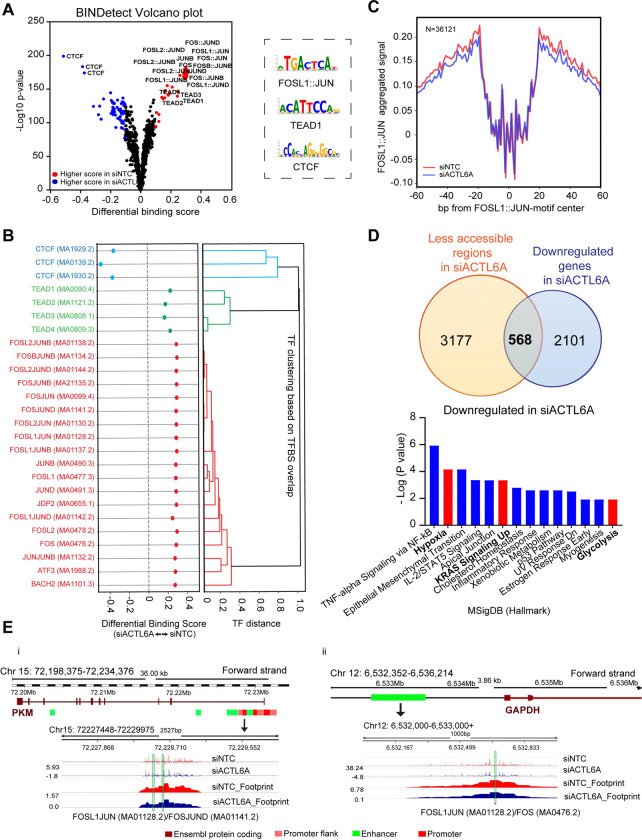
Chromatin accessibility at AP-1, TEAD, and CTCF transcription factor binding sites is regulated by ACTL6A. A) Volcano plot from BINDetect analysis using TOBIAS tool on ATAC-seq data from SCC1 cells showing differential binding scores for transcription factors (TF) motifs in control samples compared with ACTL6A knockdown. B) Differential binding scores for indicated transcription factors in top three clusters (shown separately). TF clustering is based on overlapping of TF binding sites. C) Aggregated footprinting plot, comparing SCC1 siNTC and siACTL6A samples, centered on predicted binding sites for FOSL1/JUN. D) Venn diagram (upper) showing the overlapping genes between less accessible regions from ATAC-seq and significantly downregulated genes from RNA-seq in SCC1 cells upon ACTL6A knockdown and Top-ranked Hallmark pathways (lower) downregulated in SCC1 siACTL6A cells using overlapping genes (N=568) from Venn diagram analyzed in EnrichR (p < 0.05). E) Genomic tracks for PKM (i) and GAPDH (ii) were made using GRCh38.p14 assembly in Ensembl with their corresponding corrected ATAC-seq peaks and footprints generated with TOBIAS from SCC1 ATAC-seq data comparing the control samples with ACTL6A knockdown

To assess the role of ACTL6A expression on transcription factor (TF) binding and identify mechanisms contributing to our metabolic phenotype, we performed ATAC-seq (assay for transposase-accessible chromatin) using SCC1 cells following transient ACTL6A knockdown (Extended data fig. 3A). Differential accessibility analysis showed reduced accessibility in 3176 regions and increased accessibility in 1250 regions in ACTL6A knockdown cells compared with control (Extended data fig. 3B). Then, we performed footprinting analysis using TOBIAS^42^ to predict differential TF binding and clustering. Using motifs from JASPAR^43^, we found three main clusters with differential activity following ACTL6A knockdown, with FOS::JUN (AP-1) and TEAD transcription factor clusters showing significantly increased chromatin accessibility in the siNTC samples and CCCTC-binding factor (CTCF) cluster with increased chromatin accessibility in siACTL6A samples (Fig. 5A,B and Extended Data Fig. 3A–C). To further visualize the difference in binding activity across all binding sites between ACTL6A knockdown and control cells, aggregated signal was plotted around motif center (Fig. 5C and Extended Data Fig. 3D). To study whether changes in chromatin accessibility following ACTL6A knockdown correspond with our metabolic phenotype, we conducted pathway analysis using MSigDB Hallmark on 568 overlapping genes with less accessible regions on ATAC-seq and downregulation on RNA-seq in siACTL6A samples. We found downregulation of hypoxia, KRAS signaling, and Glycolysis pathways (Fig. 5D). We investigated the regulatory regions of glycolysis genes that were significantly down-regulated with siACTL6A and found reduced activity of the FOSL1::JUN TFs on the promoter and enhancer of PKM and GAPDH, respectively (Fig. 5Ei,ii). These results indicate that ACTL6A expression maintains chromatin accessibility at AP-1 TF sites, which potentiate expression of genes involved in glycolysis.

### MAPK signaling is potentiated by coordinated up-regulation and stabilization of Ras proteins

**Figure 6.**
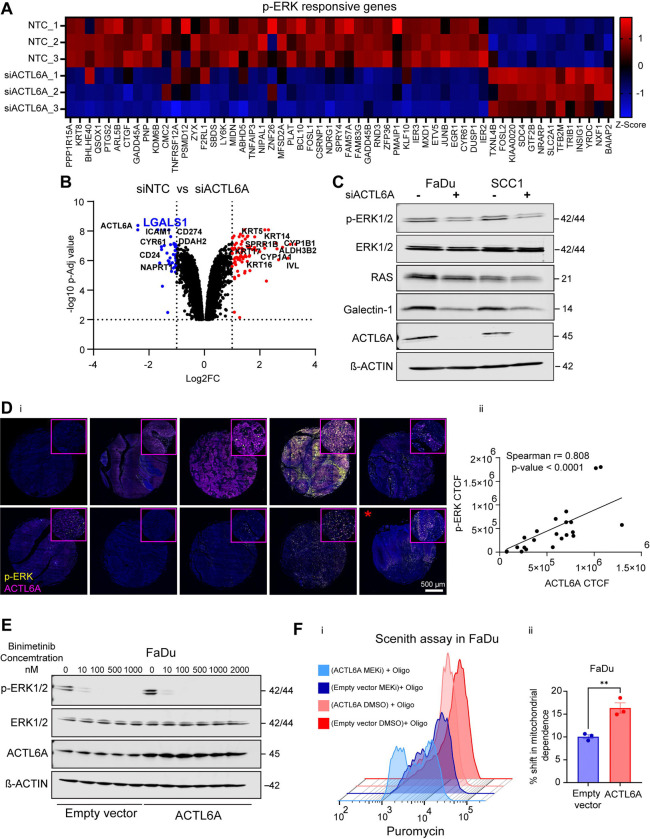
A) Heatmap showing expression of late p-ERK responsive genes using Z-scores generated from SCC1 RNA-seq data for siACTL6A and control replicates. Adj p-value <0.01 B) Volcano plot showing Log2FC of differentially expressed genes against −log10 Adj p-value in RNA-seq from SCC1 following ACTL6A transient knockdown. Blue and red colors represent downregulated and upregulated genes in siACTL6A respectively. N=3. Lof2FC< −1 and > 1 with Adj p-value <0.01 is considered significant. C) Representative Western blot images of β-actin, ACTL6A, Galectin-1, RAS, total ERK1/2, and p-ERK1/2 in FaDu and SCC1 cells following transient ACTL6A knockdown. D) Representative confocal images (i) and non-parametric spearman correlation analysis (ii) of corrected total cell fluorescence for ACTL6A (magenta) and p-ERK (yellow). Immunofluorescence staining was done on paraffin embedded freshly cut tissue array sections from either HNSCC or normal tongue tissue (red asterisk shows the normal tissue). (N=22) E) Western blots of β-actin, ACTL6A, total ERK1/2, and p-ERK1/2 in FaDu cells following ACTL6A overexpression in the presence of indicated concentrations of Binimetinib (MEK inhibitor). F) Histogram plots of Puromycin Fluorescence intensity (i) and quantitation (ii) of percentage shift in mitochondrial dependence calculated using Scenith assay. Geometric mean fluorescence intensity of Puromycin was measured in different conditions to calculate mitochondrial dependence (see method) in FaDu cells following ACTL6A overexpression and 2 hours treatment with either DMSO or 10nM Binimetinib (MEK inhibitor). N=3. Data are mean ± SEM, ** p < 0.01, unpaired t tests

Mitogen-activated protein kinase (MAPK) signaling pathway is a tightly regulated signaling cascade that induces AP-1 TF binding^44^. By integrated analysis of ATAC-seq and RNA-seq, we showed that KRAS signaling was downregulated upon ACTL6A knockdown in SCC1 cells (Figure 5D). Further analysis using RNA-seq also revealed downregulation of the majority of late p-ERK responsive genes^45^ in SCC1 cells with ACTL6A knockdown (Fig. 6A). While our ATAC-seq data supports a direct role of ACTL6A in potentiating an open chromatin configuration at AP-1 TF sites across the genome, we studied whether up-stream MAPK signaling pathways could also be affected by ACTL6A. We identified LGALS1/Galectin-1 among the top five most downregulated genes with ACTL6A knockdown (Log2FC = −1.6, Adj p-value= 8.32E-09) (Fig. 6B). Galectin-1 stabilizes RAS protein anchorage to the cell membrane, which potentiates downstream signaling through MEK-ERK^46^. We identified a TEAD1 binding site in the *LGALS1* promoter, which had significantly decreased accessibility in siACTL6A samples (Extended data Fig. 4A). Additionally, we found significantly decreased expression of *KRAS*, *NRAS*, and *HRAS* gene expression using RT-qPCR in SCC1 cells following ACTL6A knockdown relative to control and a significant correlation between ACTL6A and Ras gene expression in our DepMap dataset (Extended data Fig. 4B,C). We studied whether changes in *Ras* and *LGALS1* expression could alter phosphorylation of ERK and expression of p-ERK responsive genes. We quantified protein expression of Galectin-1, RAS, and p-ERK1/2 in FaDu and SCC1 cells following ACTL6A knockdown, which showed reduced expression of these proteins after siACTL6A treatment (Fig. 6C). To test if ACTL6A is correlated with p-ERK expression in human HNSCC tissue, we performed immunofluorescence staining for both proteins on freshly cut tissue microarray (TMA) of ten HNSCC tumors and two normal upper aerodigestive tract mucosa specimens (Fig. 6D). We found a strong, significant correlation between ACTL6A and p-ERK expression in the TMA (r = 0.808, p<0.001) (Fig. 6E). These data link ACTL6A with induction of up-stream MAPK signaling, indicating coordinated regulation of AP-1 signaling through distinct mechanisms.

Oncogenic RAS mutations are implicated in improving cancer cell survival by shifting cell metabolism to aerobic glycolysis^47^. Therefore, we hypothesized that ACTL6A’s metabolic effects are mediated through MAPK signaling and may be susceptible to MEK inhibitors. To test this, we first treated either FaDu control or ACTL6A overexpressing cells with either DMSO or 10nM Binimetinib, a small molecule inhibitor of MEK1/2, (hereafter as MEKi) for 2 hours. We selected this concentration based on the ability of MEKi in inhibiting ERK phosphorylation (Fig. 6E). We then performed SCENITH assay (Fig. 6F), which showed that addition of oligomycin resulted in a shift in both control and ACTL6A overexpressing cells in the presence of MEKi (light and dark blue in Fig. 6F), suggestive of higher mitochondrial dependency in both conditions. ACTL6A overexpressing cells (light blue) showed a significantly larger shift toward mitochondrial dependence compared to control cells (Fig 6F, right), indicating much greater sensitivity to the metabolic effects of MEKi. These data suggest MEKi may be useful in treating ACTL6A overexpressing tumors with high rates of glycolysis and hypoxia.

## Discussion

Uncovering pathways that regulate cancer cell metabolism provides insights into oncogenesis and offers therapeutic targets. As cancer cells shift metabolism from oxidative phosphorylation to aerobic glycolysis, they reduce their exposure to ROS and increase glucose turnover, both of which enhance proliferation^13^. We have discovered that expression of ACTL6A, a gene on the 3q26 amplicon amplified in HNSCC tumors, acts as a switch that suppresses mitochondrial dependency and up-regulates aerobic glycolysis, leading to decreased ROS, cisplatin resistance, and ability to grow in hypoxic environments. These effects are mediated by coordinated mechanisms that amplify ERK signaling through up-regulation of Ras and Galectin-1 as well as stabilizing chromatin accessibility at AP-1 transcription factor binding sites. This pathway can be modulated for therapeutic benefit, either through MEK inhibition or a synthetic lethal strategy targeting ACTL6A expression with inhibitors of oxidative phosphorylation. These data establish ACTL6A as a key regulator of the Warburg effect in HNSCC and propose ACTL6A inhibition, either through SWI/SNF inhibition or development of a direct inhibitor, as a potential treatment strategy for HNSCC.

The RAS/RAF/MAPK pathway has been linked to activation of glycolysis in several cancers. Notably, KRAS over-expressing models of pancreatic cancer significantly up-regulate glucose uptake and glycolysis gene expression^47^. Similarly, MEK inhibitors have been shown to significantly decrease glycolysis in a range of tumors^48^. Up-regulation of oxidative phosphorylation and mitochondrial biogenesis are key mechanisms of resistance to MAPK pathway inhibitors, which can be overcome with administration of metabolic inhibitors to rescue their anti-tumor effects^49–51^. Our study demonstrates a novel mechanism through which cancer cells up-regulate aerobic glycolysis by demonstrating an increase in chromatin accessibility at AP-1 transcription factor binding sites at glycolysis gene promoters. ACTL6A expression levels mediate access to these sites as well as regulating phosphorylation of ERK, thereby enhancing both AP-1 transcription factor activity and chromatin access. MEK inhibitors also showed a role in increased mitochondrial dependency to a much stronger degree in ACTL6A over-expressing tumors, suggesting their potential for treating a large subset of HNSCC tumors with increased ACTL6A expression. As many RAS/RAF/MAPK inhibitors are clinically approved for use in other cancers^52^, it raises the possibility of clinical trials in HNSCC after stratifying tumors using hypoxia imaging or ACTL6A expression^12,53^.

ACTL6A-mediated up-regulation of ERK signaling and chromatin accessibility of AP-1 binding sites has not previously been reported. While our ATAC-seq data recapitulated increased TEAD/YAP chromatin accessibility with increasing ACTL6A expression levels^22,23^, AP-1 signaling is likely to be more important for maintenance of the metabolic phenotype in our study. YAP is known to promote cell proliferation and oncogenesis in HNSCC^54^, but its activation is generally modulated by cellular energy states rather than having a direct impact on them^55^. For instance, several studies have shown that high glucose turnover and aerobic glycolysis activate YAP while energy stress leads to its inhibition^56–58^. There are relatively few studies linking SWI/SNF and MAPK pathways, but two recent papers support SWI/SNF potentiating MAPK signaling. Notably, Wolf et al (2023) showed that multiple SWI/SNF subunits are enriched at AP-1 transcription factor binding sites and cooperate to maintain chromatin accessibility^59^. Miguel et al (2023) showed that resistance to tyrosine kinase inhibitors in lung cancer was mediated by up-regulation of SWI/SNF chromatin binding at downstream MAPK genes, which could be overcome using inhibition of the SWI/SNF subunits SMARCA2 and SMARCA4^60^. ACTL6A is also a component of other chromatin remodeling complexes, including the histone acetyltransferase complex TIP60^61^, which may be important for promoting expression of Ras genes up-stream of ERK. There are currently no CUT&RUN or ChIP-seq datasets available of ACTL6A chromatin occupancy and these follow-up studies are needed to demonstrate the extent to which SWI/SNF, TIP60, and other chromatin complexes contribute to oncogenic phenotypes in HNSCC.

Modifying ACTL6A expression had significant effects on mitochondrial dependency in HNSCC cells, with low levels of ACTL6A abrogating hypoxic cell growth as well as sensitizing cells to cytotoxicity using the oxidative phosphorylation inhibitor IACS-010759. Increased mitochondrial dependence after ACTL6A knockdown is likely mediated by inability to up-regulate glycolysis in HNSCC cells rather than a change in mitochondrial function. Both MAPK signaling and SWI/SNF have been linked to changes in mitochondrial metabolism in other cancers. In melanoma, BRAF inhibition was shown to up-regulate oxidative phosphorylation through increased PGC1α and MITF expression^62^. SMARCA4 deficient lung cancers were also shown to up-regulate oxidative phosphorylation through activation of PGC1α^63^; however, PGC1α and MITF did not have detectable expression in SCC1 cells even after ACTL6A knockdown (see RNA-seq dataset), suggesting other transcriptional mediators may be at play. Finally, on our Seahorse analysis, there was a much greater decrease in basal ECAR than increase OCR in SCC1 cells with ACTL6A knockdown, suggesting ACTL6A levels primarily impact glycolysis. The extent to which ACTL6A acts directly on mitochondria needs further exploration and will be the subject of follow-up studies.

IACS-010759 was shown to significantly suppress growth of the treatment resistant MOC2 cell line after ACTL6A knockdown. This is similar to its reported efficacy for SMARCA4 mutated lung cancers reported by Deribe et al. (2018)^63^, suggesting oxidative phosphorylation inhibitors generally have potential for treatment of SWI/SNF depleted tumors. IACS-010759 has undergone phase I clinical trials in a wide range of solid tumors and acute myeloid leukemia (AML) with some reports of efficacy but significant dose limiting toxicities, including lactic acidosis and peripheral neuropathies^39^. Notably, patients were treated without screening for mutations or baseline metabolic status of tumors. While clinical development of IACS-010759 is limited by toxicity, the results in this study raise the question of whether SWI/SNF deficient tumors may be more susceptible to its effect in a lower therapeutic window where adverse effects are more tolerable. Additionally, inhibitors of other components of aerobic metabolism may also be studied. CPI-613 is a tricarboxylic acid (TCA) cycle inhibitor that has been safely delivered to humans in a clinical trial for pancreatic adenocarcinoma^64^. Gamintrinib is a mitochondrial matrix inhibitor with potentially improved toxicity profile compared to IACS-010759 under clinical trial for solid tumors^65^. The extent to which SWI/SNF deficient malignancies respond to metabolic inhibitors will be the subject of further study.

In conclusion, we have identified ACTL6A, a SWI/SNF subunit over-expressed in a large subset of HNSCC tumors, as a key mediator of the Warburg effect. ACTL6A levels act as a switch that regulate mitochondrial dependence and hypoxic cell growth. Modulating these levels sensitizes cancer cells to the tumor killing effects of oxidative phosphorylation inhibitors, suggesting a new line of treatment for HNSCC. These effects are induced by coordinated up-regulation of AP-1 signaling through induction of Ras expression and the Ras accessory protein Galectin-1 as well as regulating chromatin access at AP-1 transcription factor binding sites. Together, these mechanisms drive aerobic glycolysis and oncogenic phenotypes. Targeting ACTL6A and its downstream pathways may have therapeutic benefit for HNSCC.

## Materials and Methods

### Cell lines and culture conditions

FaDu (HTB-43 ^™^) and UM-SCC1(SCC070) cell lines were purchased from ATCC (American Type Culture Collection) and Sigma-aldrich respectively. MOC2 (Mouse oral squamous cell carcinoma) cell line was a gift from Ravi Uppaluri. All cell lines were cultured in DMEM-F12 supplemented with L-glutamine, HEPES, 10% FBS, and 100 IU/ml penicillin 100μg/ml streptomycin and incubated at 37°c in a 5% CO2 incubator. Cell line authentication was done by ATCC cell authentication service using STR profiling. Cells were tested frequently for the presence of Mycoplasma using Bulldog Bio Inc E-MYCO mycoplasma PCR kit.

### Transwell migration assay

10 μg/ml fibronectin in sterile DI water was added to transwell inserts with 6.5mm diameter and 8μm pore size and incubated at 37°c for 2 hours. FaDu and SCC1 cells were suspended in serum free media at 500000/ml density. After removing fibronectin solution from the inserts, 200 μl cell suspension was added to the upper chamber while lower chamber was filled with 600 μl complete growth media. Cells were incubated for 24 hours at 37°c in a 5% CO2 incubator. Then, medium was removed and inserts were washed gently with PBS twice and non-invaded cells were removed from the membrane by cotton tipped applicators. Next, the cells were fixed using 10% NBF (neutral buffered formalin) for 5 minutes at RT. Then the cells were washed with PBS and permeabilized with 100% ethanol for 20 minutes and stained with 0.5% crystal violet in 25% methanol in DI water for 15 minutes. Next, transwells were washed with DI water and air dried at RT overnight. Then membranes were cut and mounted on microscope slides with mounting medium. Imaging was done using ZEISS Axio Imager 2.

### Resazurin proliferation assay

FaDu and SCC1 cells were cultured in 96 well plates at density of 2500 per well and incubated at 37°c in a 5% CO2 incubator. After either 24 or 48 hours the media was removed from the cells and 100 μl resazurin solution (diluted 1 to 10 in growth medium) was added to each well and incubated for 4 hours at 37°c in CO2 incubator. Next, absorbance was measured at 600 and 570 nm using Multiskan skyhigh plate reader (Thermofisher) and background absorbance at 600 nm was subtracted from resorufin absorbance at 570nm.

### SRB (Sulforodhamine b) proliferation assay

FaDu and SCC1 cells were cultured in 96 well plates at density of 2500 per well and incubated at 37°c in a 5% CO2 incubator. After either 24 or 48 hours the media was removed and cells were fixed with 100 μl of 10% TCA (trichloroacetic acid) for 10 minutes at 4°c. Then, TCA was removed and cells were washed with DI water 3 times. Next, 100 μl SRB solution (0.2% SRB in 1% acetic acid) was added to each well and incubated for 10 minutes at RT. Then the cells were washed with 1% acetic acid 3 times and the plate was air dried overnight at RT. Next, 100μl Tris base solution (20 mM, PH: 10) was added to each well and incubated on a shaker for1 hour at RT. Absorbance was measured at 510nm using MultiSkan Skyhigh plate reader.

### ACTL6A transient Knockdown

Reverse Transient knockdown was done using ON-TARGET plus human ACTL6A SMART pool siRNAs (l-008243-00-0005, Dharmacon). Briefly, either ACTL6A or non-targeting control siRNA (ON-TARGET plus Non-targeting Pool, D-0001810-10-05, Dharmacon) was added at 100 nM concentration to 150 μl serum free OPTIMEM medium. Next, 10 μl transfection reagent (X-tremeGENE^™^ 360, Sigmaaldrich) was mixed with OPTIMEM medium and incubated at RT for 30 minutes. Then the mixture was added dropwise to a 6 well plate contained 1.5 ml growth OPTIMEM with 10% FBS. Next FaDu and SCC1 cells were transferred to the wells at density of 200,000 cells/well. After 72 hours the cells were collected for downstream experiments and analysis.

### ACTL6A stable knockdown

Two individual SMARTvector mouse lentiviral shRNAs (Dharmacon, V3SM11241-235868768 and V3SM11241-237525934) against mouse actl6a were used for stable actl6a knockdown in MOC1 cells. Briefly, HEK293T cells were cultured in a 100mm culture plate in DMEM-F12 supplemented with 10% FBs and 100 IU/ml penicillin 100μg/ml streptomycin. The next day, 10 μg shactl6a plasmid, 5 μg of each packaging vector (pMD2.G and psPAX2), and 50 μg PEI were mixed with 500 μl serum free OPTIMEM medium and incubated at RT for 30 minutes. Then the mixture was added to the cells dropwise. The next day, the medium was replaced with fresh complete growth medium. Viral particles containing media was collected at 48 and 72 hours, and filtered by 0.45μm PES syringe filter then concentrated using Amicon Ultra centrifugal filter at 3000 rpm for 10–20 minutes at 4°c. To transduce MOC1 cells, they were cultured in 6 well plate for 24 hours, then concentrated viral particles were added to their medium with 10 μg/ml polybrene. After 48 hours, selection was done using 2 μg/ml Puromycin.

### ACTL6A overexpression

Lentiviral vector N106 cloned with ACTL6A cDNA, a gift from Dr. Crabtree’s lab, was used for ACTL6A overexpression in FaDu cells. Lentiviral production and transduction were done as described in “ACTL6A stable knockdown” and 10 μg/ml Blasticidin was used for selection.

### Mitochondrial labeling

pLV-mitoDsRed lentiviral vector (a gift from Pantelis Tsoulfas (Addgene plasmid # 44386; http://n2t.net/addgene:44386; RRID: Addgene 44386)) was used to label mitochondria. In brief, FaDu and SCC1 were cultured in 6 well plates until about 50% confluent. Then lentiviral particles carrying pLV-mitoDsRed vector and 10 μg/ml Polybrene were added to the cells and incubated for 48 hours. Next DsRed positive cells were sorted using BD FACSAria sorter. Labeled FaDu and SCC1 cells were cultured in Thermo Scientific^™^ Nunc^™^ Lab-Tek^™^ II Chamber Slides followed by ACTL6A transient knockdown for 72 hours. Then the cells were washed with cold PBS twice and fixed with 10% NBF for 15 minutes. After washing with PBS for 3 times, permeabilization was done using 0.03% triton 100x in PBS for 10 minutes. The chambers were washed with PBS and mounted with prolong gold antifade mounting reagent with DAPI (Invitrogen; P36941). Imaging was done using ZEISS LSM700 confocal microscope. CTCF (corrected total cell fluorescence) for DsRed in each field was measured in Fiji 2.16.0 and normalized to the total number of cells per field.

### MitoTracker Deep Red FM and MitoSOX Red staining

MitoTracker Deep Red FM (MTDR) and MitoSOX (MedChemExpress; Cat. No.: HY-D1783 and HY-D1055 respectively) staining were done following manufacturer’s protocol. Briefly, FaDu and SCC1 cells, Following ACTL6A transient knockdown, were incubated with either 100nM MTDR or 5μM MitoSOX in Serum-free medium for 30 minutes at RT. Then, cells were washed with medium twice for 5 minutes. Flowcytometry was done using BD FACSymphony A5. FlowJo-V10 was used for analysis.

### Seahorse XF Real-Time ATP Rate Assay

Real-time cellular metabolism was analyzed using an Agilent Seahorse XFe96 analyzer (Agilent Technologies, USA) to measure oxygen consumption rate (OCR), extracellular acidification rate (ECAR), total ATP production rate, and the relative contributions of glycolytic and mitochondrial pathways. Briefly, SCC1 cells were seeded at 20,000 cells/well in a Seahorse XF96 culture microplate and maintained in their growth medium for 24 hours at 37°C under 5% CO_2_. A day prior to the experiment, the Seahorse XF96 sensor cartridge was hydrated with 200 ul water and incubated in a non-CO2 incubator at 37°C. Two hours before the experiment, the water was replaced with a Seahorse XF calibrant, and the cartridge re-incubated at 37°C (non-CO2). On the day of the experiment, Seahorse XF DMEM (pH = 7.4) was supplemented with 0.5 mM pyruvate, 2.5 mM glutamine and 10mM glucose (Agilent, USA). Culture medium was replaced with 180 ul/well of supplemented Seahorse XF DMEM medium. Cells were incubated in a non-CO2 incubator at 37°C for 1 hour. During this incubation period, oligomycin and Rotenone/Antimycin were prepared in the Seahorse medium to achieve final concentration of 20 uM upon injected. The hydrated sensor cartridge was loaded with 20 ul and 22 ul of Oligomycin and rotenone/antimycin A respectively in ports A and B and calibrated for 30 minutes. The calibration plate was replaced with the cell culture plate upon calibration completed. Measurements followed sequential injection of inhibitors using the default program. Cell numbers were quantified post-assay using SRB assay for OCR/ECAR normalization. Data analysis was done using Wave 2.6.3 software.

### SCENITH assay

SCENITH assay was performed according to the protocol outlined by Arguello et al. (2020)^35^. Briefly, 72 hours after transient ACTLA6 knockdown, the cells were treated with DMSO, 2μM oligomycin (O), 100 mM 2-Deoxy-D-glucose (2DG) (Medchemexpress, Cat. No: HY-13966) or combination of oligomycin and 2DG (2DGO). After 15 minutes incubation, Puromycin was added to the cells at final concentration of 10μg/ml and incubation continued for another 30 minutes. Then the cells washed with PBS and stained withZombie Green^™^ Fixable Viability dye (Biolegend, Cat. No: 423111). Next, the cells were stained with Alexa Fluor^®^ 647 anti-Puromycin Antibody (Biolegend, Cat. No: 381508) using eBioscience^™^ Foxp3 / Transcription Factor Staining Buffer Set (Invitrogen, Cat. No: 00-5523-00). Incubation with anti-puromycin antibody was done in the permeabilization buffer at 4°c for 1 hour in dark. Flow cytometry was done using BD FACSymphony A5. Geometric mean Fluorescence intensity (gMFI) of Puromycin was measured in all samples using FlowJo-V10. To calculate mitochondrial dependence, glycolytic capacity, glucose dependence, and fatty acid and amino acid oxidation capacity, following formulas were used:

Mitochondrialdependence(%)=100*((DMSO−O)/(DMSO−2DGO))

GlycolyticCapacity(%)=100−Mitochondrialdependence(%)

Glucosedependence(%)=100*((DMSO−2DG)/(DMSO−2DGO))

fattyacidandaminoacidoxidationcapacity(%)=100−Glucosedependence(%)

### IACS-010579 cytotoxicity measurement

To determine the cytotoxicity of IACS-10759, MOC2 and FaDu cells were cultured in 96 well plates at density of 500 and 2500 cells per well respectively. After 24 hours incubation, IACS was added to the cells at indicated concentrations (Fig 6A) and incubated for 5 days. The medium was replaced with fresh medium contained IACS at day 3. SRB assay was done at day 5 as previously described.

### Bulk ATAC-seq

ATAC-seq library prep was done using ATAC-seq Kit (Active Motif, Cat. No: 53150). Briefly, 100,000 SCC1 cells were collected after 72 hours siRNA transfection. Nuclei preparation, tagmentation reaction and purification, PCR amplification of tagmented DNA were done according to manufacturer’s protocol. Libraries size distribution was analyzed using Agilent 2100 bioanalyzer. Libraries were sequenced in 50 bp paired-end reads on Illumina NovaSeq X Plus at MedGenome Inc. Analysis was done using nf-core ATAC-seq pipeline (nf-core/atacseq: 2.1.2)^66^. In brief, alignment to human reference genome (GRCh38) was done using Bowtie2 aligner (2.4.4). Narrow peaks were called by MACS2 (2.2.7.1) and differential accessibility analysis was conducted with DESeq2 (1.28.0). Foot printing analysis was performed using TOBIAS 0.13.3.

### RNA-seq

Total RNA extraction was done using Trizol/chloroform method. SCC1 cells were collected following transient ACTLA6 knockdown in 1.5 ml microcentrifuge tubes. 500 μl Trizol (Invitrogen, Cat. No: 15596026) was added to the cell pellets then phase separation was done by adding 100 μl Chloroform to each tube. The mixture was incubated for 5 minutes at RT followed by centrifugation at 12000g for 15 minutes at 4°c. Top phase was transferred carefully to a new tube and 500 μl Isopropanol was added to precipitate RNA. After 10 minutes incubation at RT, the samples were centrifuged at 12000g for 20 minutes at 4°c. Next, the supernatant was discarded and washing was done with 75 % ice-cold Ethanol twice. RNA was dried at Rt for 10 minutes and suspended with Nuclease free ultrapure water. Libraries were made using Illumina^®^ Stranded mRNA Prep kit (20040534). Sequencing was performed by MedGenome Inc on Illumina NovaSeq X Plus sequencing system. Data analysis was done using nf-core RNA sequencing analysis pipeline (nf-core/rnaseq: version 3.14.0). Differential gene expression analysis was done using DESeq2.

### Immunofluorescence staining of tissue sections (Paraffin embedded)

Fresh cut human HNSCC tissue array slides (HN242c) were purchased from Tissue Array (Derwood, MD). Tissue staining was performed using ACTL6A and p-ERK antibodies. Briefly, slides were baked at 60°C for 2 hours. Then the slides were washed in xylene for 10 minutes (3x) followed by washing in 100%, 95%, 70% ethanol, and DI water for 5 minutes each. Antigen retrieval was done in 10mM Sodium Citrate (PH=6) for 15 minutes in a pressure cooker. Then the slides were cooled down in the same buffer on ice for 30 minutes and washed with 1x PBS twice for 10 minutes. Permeabilization was done in 1x PBS/0.2% gelatin/0.25% Triton buffer for 20 minutes. To block non-specific binding, Tissue sections were incubated in 0.5% BSA (dissolved in the permeabilization buffer) for 1 hour at RT. Primary antibodies (diluted in 1% blocking buffer (for dilutions refer to table 2)) added on tissue sections and incubated in a humidity chamber overnight at 4°C. Next, the slides were washed in PBS twice and permeabilization buffer once for 10 minutes each. Sections were then incubated with secondary antibodies (1 to 400 dilution in 1% permeabilization buffer) for 1 hour at RT. Sections were washed with PBS twice and mounted with ProLong^™^ Gold Antifade Mountant with DNA Stain DAPI (Invitrogen, Cat No: P36931). Imaging was done using ZEISS LSM700 confocal microscope. Image analysis was performed using Fiji 2.16.0.

### Animal studies

All in vivo experiments were done in accordance with the Institutional Animal Care and Use Committee at Stanford University. NOD.Cg-Rag1^tm1Mom^ Il2rg ^tm1Wjl^/Sz (RRID:IMSR_JAX:007799) mice were injected subcutaneously with 500,000 MOC2 shNTC or shActl6a cells. When average tumor size reached 200 mm^3^, IACS-10759 (MedChemExpress, Cat. No.: HY-112037) or vehicle (10% DMSO/40% PEG300/5% Tween-80/45% Saline) was given to mice by oral gavage daily at 10 mg/kg. Tumor width and length were measured by Caliper every 3–4 days and volume was calculated as follows: Tumor volume = Length × width^2^ × 0.5. Mice were euthanized when tumors reached 1000 mm^3^ within one treatment group and tumor tissue was collected for further analysis.

### qRT-PCR

RNA isolation was done as described in (*Bulk RNA sequencing*). cDNA was synthesized using HiScript III 1st Strand cDNA Synthesis Kit (Vazyme, Cat. No: R312-01) following manufacturer’s protocol. qPCR reactions were done using Taq Pro Universal SYBR qPCR Master Mix (Vazyme, Cat. No: Q712-02) in QuantStudio 7 Pro Real-Time PCR System. mRNA expression was calculated relative to RPL13a.

### Western blot

Cells were washed with cold PBS and lysed using RIPA lysis buffer (Miliporesigma: 20108) supplemented with phosphatase/protease inhibitors cocktail for 30 minutes on ice. Then the lysate was centrifuged at 12000 g for 10 minutes at 4°c. Supernatant was collected and protein concentration was measured using Pierce^™^ BCA Protein Assay Kit (Thermo Scientific: 23227). 20 μg protein was boiled with Laemmli SDS sample buffer for 5 minutes at 95°c and electrophoresis was done using Novex^™^ Tris-Glycine Mini Protein Gels, 4–12% (Invitrogen^™^: XP04205BOX). Transfer was performed on iBlot 3 Western Blot Transfer System. Then the membrane was incubated in Intercept^®^ (PBS) Blocking Buffer (LI-COR: 927-70001) at RT for 1 hour followed by overnight incubation in primary antibody at 4°c. the next day, membrane was washed in TBST buffer (Tris buffered saline + 0.1% Tween 20) three times for 10 minutes followed by incubation in secondary antibody at RT for 1 hour. Membrane imaging and analysis was done using Odyssey CLx Imager and image studio software respectively.

### DepMap studies

We used the Dependency Map Project (DepMap) repository of cell line encyclopedia to search for oral and larynx SCC subsites, which are generally HPV (−). There were 62 cell lines (Extended Table 2) for which RNA-seq data was downloaded. Expression data for ACTL6A, glycolysis genes, and Ras genes was exported to GraphPad and a Spearman r co-efficient and p-value was calculated.

## Supplementary Material

Supplement 1 Extended Data Table 1. PCR primers
Extended Data Table 2. DepMap Cell Lines

Supplement 2 Extended Data Figure 1.
Heatmaps of indicated Buffa hypoxia genes for each biological replicates from SCC1 following transient ACTL6A knockdown using Z-scores calculated from RNA-seq data. N=3

Supplement 3 Extended Data Figure 2.
A) Western blot images of β-actin and ACTL6A in FaDu cells after transduction with ACTL6A expressing lentiviral particles.
B) Stable knockdown of MOC2 cells using shRNA against ACTL6A leads showing RNA levels by RT-qPCR (left) and protein levels by Western blot (right).

Supplement 4 Extended Data Figure 3.
A) Principal component analysis on top 500 genes from ATAC-seq in SCC1 siNTC (control) and siACTL6A (knockdown) three biological.
B) Volcano plot mapping log2FC of differential accessible regions from ATAC-seq data in siACTL6A over siNTC in SCC1 cells. Blue color shows less accessible regions in siACTL6A (N=3176) and more accessible regions are shown with red color (N=1250).
C) Differential binding score for the top three significant TF clusters generated using TOBIAS tool from SCC1 comparing siNTC with siACTL6A samples.
D) Aggregated footprinting plot, comparing SCC1 siNTC and siACTL6A samples, centered on predicted binding sites for TEAD1 (left) and CTCF (right).
E) There were 51 over-lapping regions with up-regulated expression on RNA-seq and increased chromatin accessibility on ATAC-seq in siACTL6A relative to controls. Reactome was used for a pathway analysis on these regions and significant (p < 0.05) pathways are shown.

Supplement 5 Extended Data Fig. 4
A). Genomic track for LGALS1 was made using GRCh38.p14 assembly in Ensembl with their corresponding corrected ATAC-seq peaks and footprints generated with TOBIAS from SCC1 ATAC-seq data comparing the control samples with ACTL6A knockdown.
B). Relative mRNA expression of ACTL6A, HRAS, NRAS, and KRAS over RPL13A (housekeeping gene). N=3. Data are mean ± SEM, * p < 0.05, ** p < 0.01, **** p <0.0001, multiple unpaired t tests.
C). Correlation analysis between ACTL6A and RAS genes in 62 HNSCC cell lines from DepMap.

## Data Availability

The ATAC-seq and RNA-seq datasets generated for siACTL6A and control SCC1 cells have been deposited in the GEO database under accession code GSE305164. Source data related to the experiments in this protocol have been deposited to the Dryad data repository using a partnership with Stanford University (DOI: 10.5061/dryad.1rn8pk16b).
