# Supplementary material for: ACTL6A regulates the Warburg effect through coordinated activation of AP-1 signaling in head and neck squamous cell carcinoma
Source: bioRxiv. 2025 Sep 4:2025.09.02.671886. Preprint. [Version 1] doi: 10.1101/2025.09.02.671886 (PMC12424702; doi:10.1101/2025.09.02.671886)
Supplement: Supplement 1 — Extended Data Table 1. PCR primers Extended Data Table 2. DepMap Cell Lines [file media-1.docx]

**Extended Data Table 1.**

| **qRT-PCR Primers** | **Sequence (5’ to 3’)** |
| --- | --- |
| KRAS F human | TGAGAGAGATCCGACAATACAGAT |
| KRAS R human | GCATCATCAACACCCAGATTAC |
| HRAS F human | ACGCACTGTGGAATCTCGGCAG |
| HRAS R human | TCACGCACCAACGTGTAGAAGG |
| NRAS F human | GAAACCTCAGCCAAGACCAGAC |
| NRAS R human | GGCAATCCCATACAACCCTGAG |
| RPL13A F human | CTCAAGGTGTTTGACGGCATCC |
| RPL13A R human | TACTTCCAGCCAACCTCGTGAG |
| **Mouse shRNAs** | **Gene target sequence** |
| Actl6a | CAAGTTACTGCCTATGCTT |
|  | CATACAAGATGCATGTCAA |
| **Human siRNAs (pool)** | **Target sequence** |
| ACTL6A | GAACGGAGGUUUAGCUCAU |
|  | CCUACUACAUAGAUACUAA |
|  | GUAAAGGGGUUAUCAGGAA |
|  | UGGGAUAGUUUUCCAAAGCUA |
| Non-Targeting | GAACGGAGGUUUAGCUCAU |
|  | CCUACUACAUAGAUACUAA |
|  | GUAAAGGGGUUAUCAGGAA |
|  | UGGGAUAGUUUUCCAAAGCUA |

**Extended Table 2. DepMap cell lines**

| **Oral and Larynx SCC cell lines** | |
| --- | --- |
| SCC9 | UPCISCC152 |
| SCC25 | UPCISCC154 |
| BICR31 | BICR78 |
| SCC4 | H103 |
| SCC15 | H157 |
| BICR6 | BICR3 |
| HSC2 | H314 |
| SNU46 | H357 |
| BICR16 | H376 |
| CAL33 | H413 |
| HSC4 | KON |
| BHY | KOSC2 |
| SNU1076 | OSC19 |
| PECAPJ34 | OSC20 |
| SNU1041 | UPCISCC026 |
| PECAPJ15 | UPCISCC029A |
| YD8 | UPCISCC040 |
| SNU1066 | UPCISCC072 |
| SNU899 | UPCISCC074 |
| SNU1214 | UPCISCC099 |
| YD10B | UPCISCC111 |
| PECAPJ41 | UPCISCC114 |
| PECAPJ49 | UPCISCC116 |
| A253 | UPCISCC131 |
| BICR56 | UPCISCC172 |
| HSC3 | UPCISCC200 |
| BICR22 | SAS |
| CAL27 | CA922 |
| YD15 | HSQ89 |
| FADU | HO1U1 |
| UPCISCC090 | UCSFOT1109 |
