# Supplementary figures and images for: ACTL6A regulates the Warburg effect through coordinated activation of AP-1 signaling in head and neck squamous cell carcinoma
Source: bioRxiv. 2025 Sep 4:2025.09.02.671886. Preprint. [Version 1] doi: 10.1101/2025.09.02.671886 (PMC12424702; doi:10.1101/2025.09.02.671886)

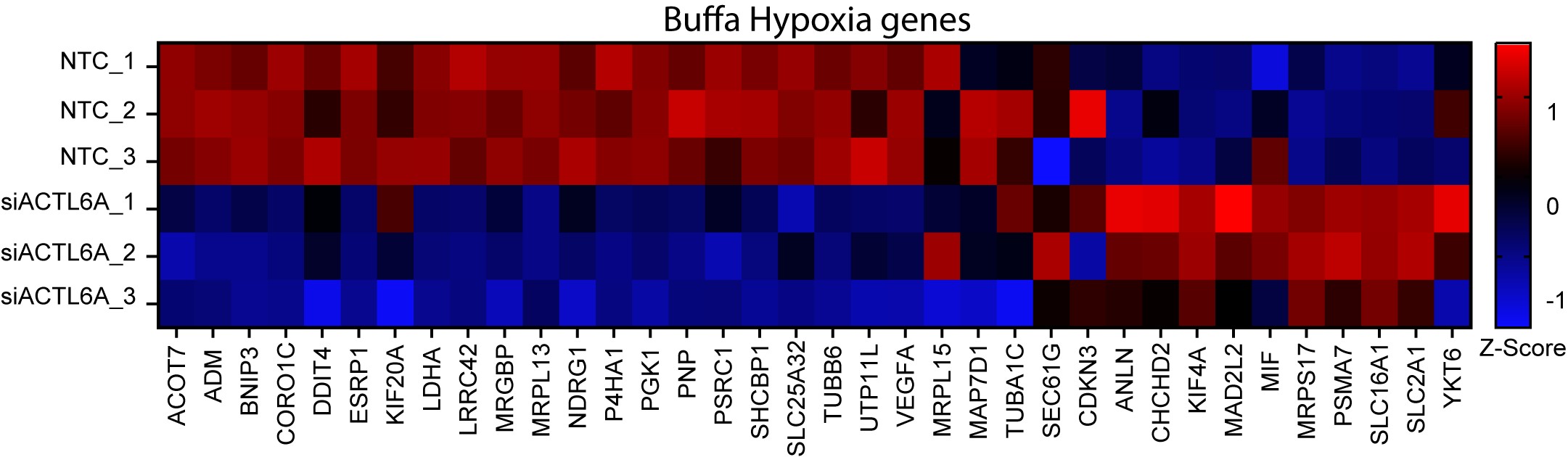

Supplement: Supplement 2 — Extended Data Figure 1. Heatmaps of indicated Buffa hypoxia genes for each biological replicates from SCC1 following transient ACTL6A knockdown using Z-scores calculated from RNA-seq data. N=3 [file media-2.jpg]

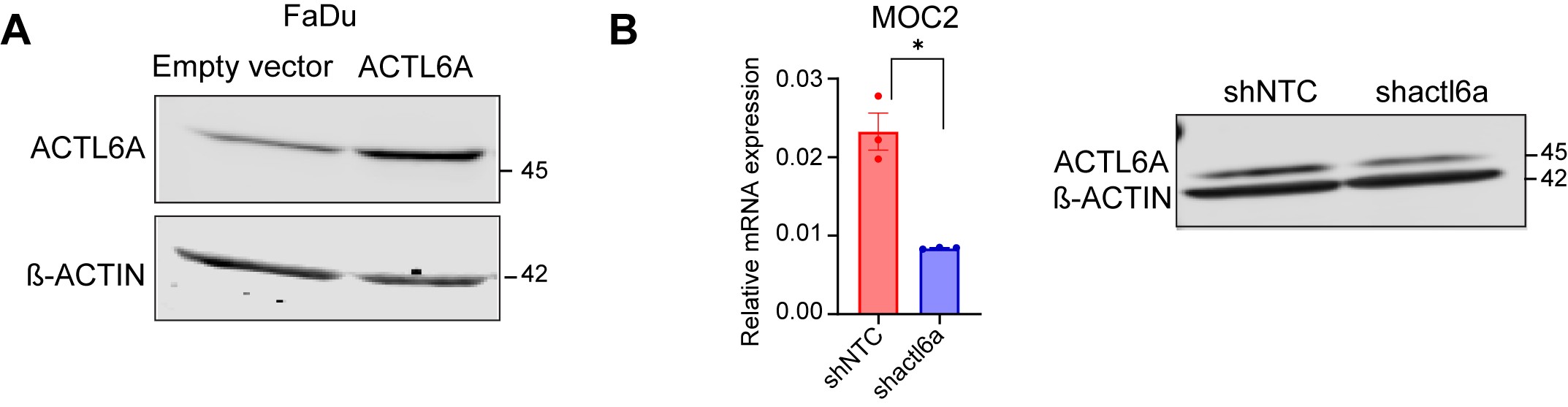

Supplement: Supplement 3 — Extended Data Figure 2. A) Western blot images of β-actin and ACTL6A in FaDu cells after transduction with ACTL6A expressing lentiviral particles. B) Stable knockdown of MOC2 cells using shRNA against ACTL6A leads showing RNA levels by RT-qPCR (left) and protein levels by Western blot (right). [file media-3.jpg]

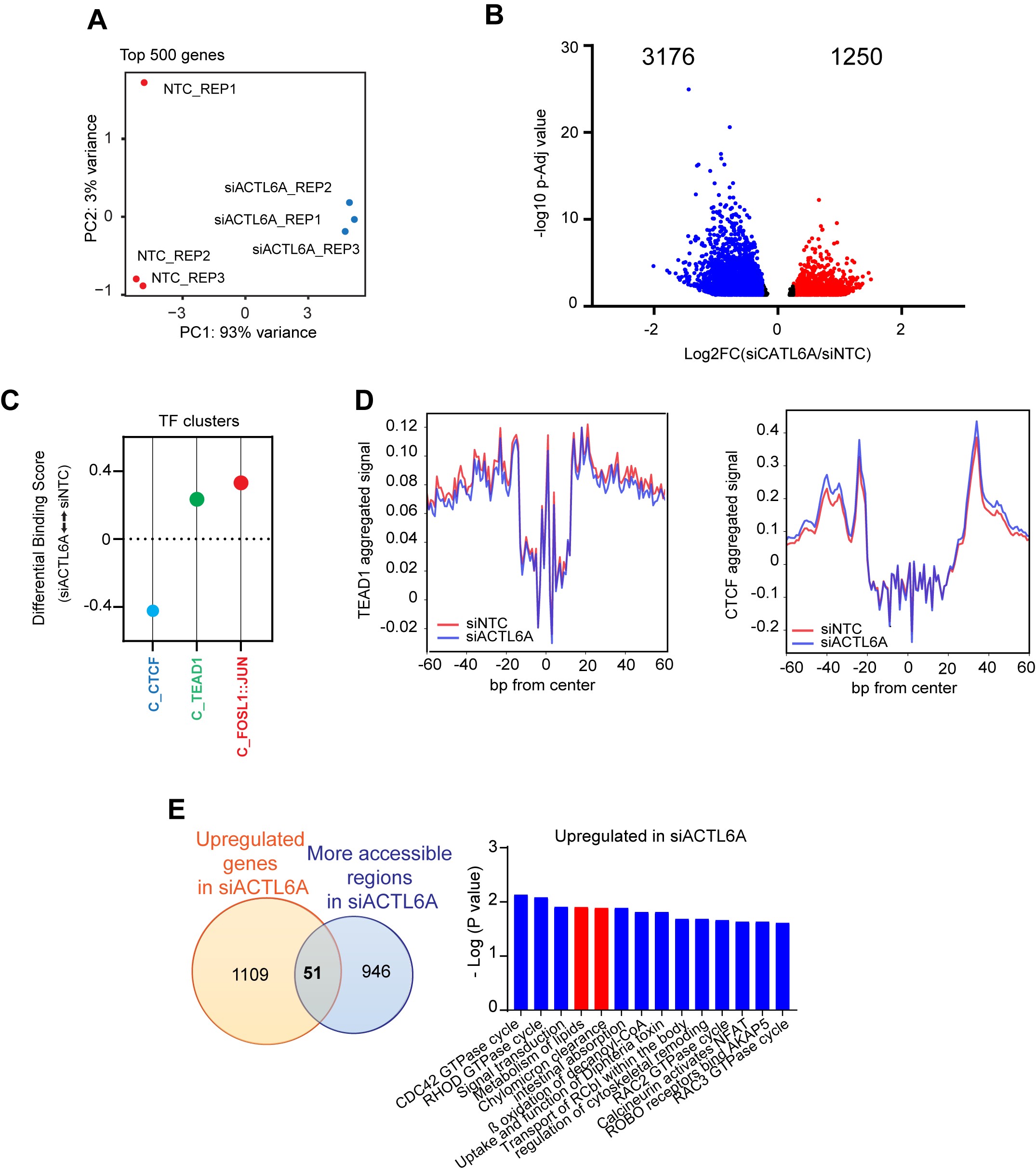

Supplement: Supplement 4 — Extended Data Figure 3. A) Principal component analysis on top 500 genes from ATAC-seq in SCC1 siNTC (control) and siACTL6A (knockdown) three biological. B) Volcano plot mapping log2FC of differential accessible regions from ATAC-seq data in siACTL6A over siNTC in SCC1 cells. Blue color shows less accessible regions in siACTL6A (N=3176) and more accessible regions are shown with red color (N=1250). C) Differential binding score for the top three significant TF clusters generated using TOBIAS tool from SCC1 comparing siNTC with siACTL6A samples. D) Aggregated footprinting plot, comparing SCC1 siNTC and siACTL6A samples, centered on predicted binding sites for TEAD1 (left) and CTCF (right). E) There were 51 over-lapping regions with up-regulated expression on RNA-seq and increased chromatin accessibility on ATAC-seq in siACTL6A relative to controls. Reactome was used for a pathway analysis on these regions and significant (p < 0.05) pathways are shown. [file media-4.jpg]

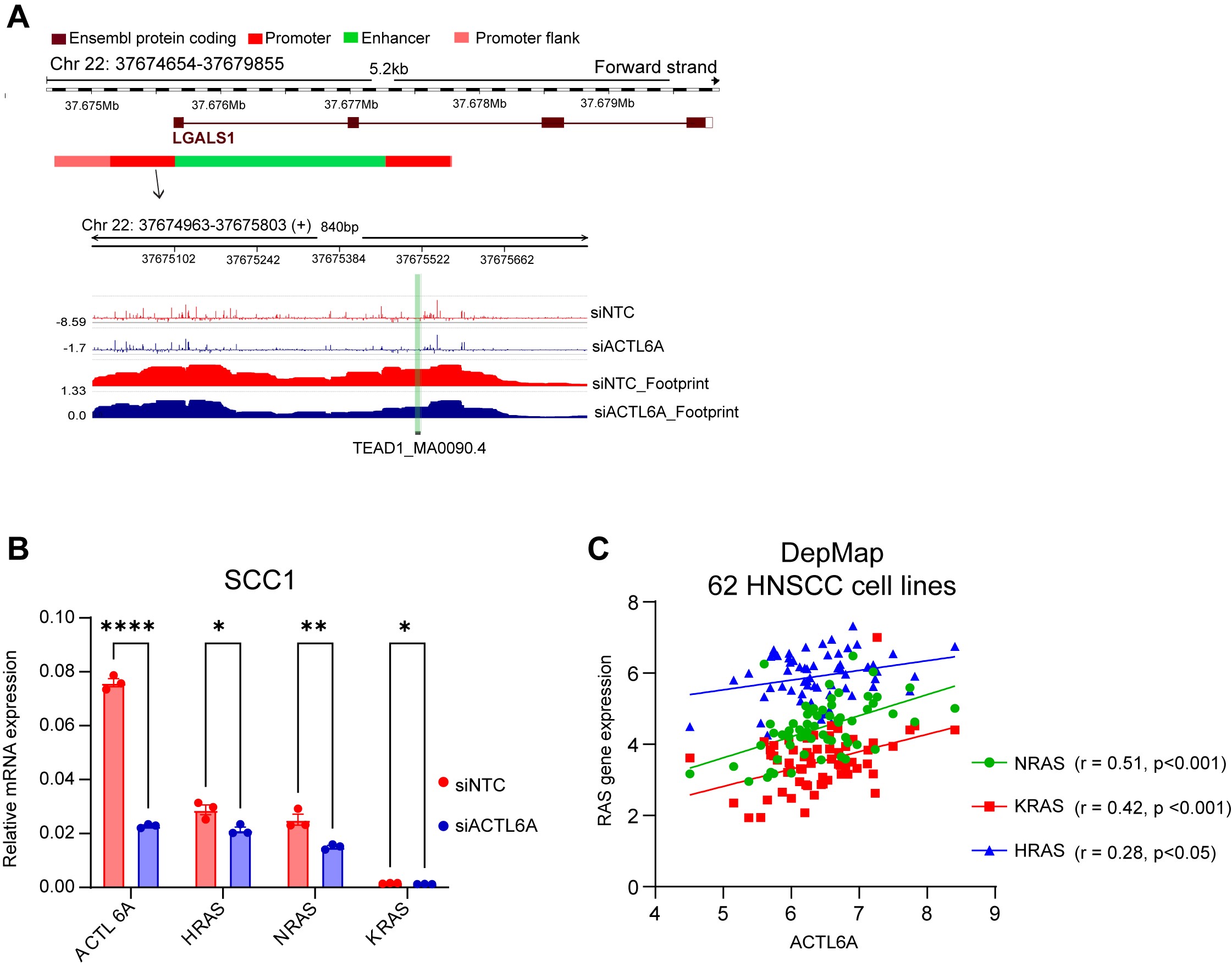

Supplement: Supplement 5 — Extended Data Fig. 4 A). Genomic track for LGALS1 was made using GRCh38.p14 assembly in Ensembl with their corresponding corrected ATAC-seq peaks and footprints generated with TOBIAS from SCC1 ATAC-seq data comparing the control samples with ACTL6A knockdown. B). Relative mRNA expression of ACTL6A, HRAS, NRAS, and KRAS over RPL13A (housekeeping gene). N=3. Data are mean ± SEM, * p < 0.05, ** p < 0.01, **** p <0.0001, multiple unpaired t tests. C). Correlation analysis between ACTL6A and RAS genes in 62 HNSCC cell lines from DepMap. [file media-5.jpg]
